# Integrating trust into artificial intelligence for medicine: using diabetes as the exemplar disease

**DOI:** 10.1186/s12967-026-07774-2

**Published:** 2026-02-25

**Authors:** Mandy M. Shao, Agatha F. Scheideman, David Kerr, Tien Y. Wong, Juan Espinoza, Shahid N. Shah, Mohammed E. Al-Sofiani, Ashley N. Beecy, Dieter Bruno, Elizabeth Healey, Nestoras Mathioudakis, Bin Sheng, Michael P. Snyder, Yih Chung Tham, David C. Klonoff

**Affiliations:** 1 Diabetes Technology Society, 845 Malcolm Road, Suite 5, Burlingame, CA 94010 USA; 2 Center for Health Systems Research, Sutter Health, Santa Barbara, CA USA; 3 Tsinghua Medicine, Beijing Visual Science and Translational Eye Research Institute (BERI), Beijing Tsinghua Changgung Hospital Eye Center, Tsinghua University, Beijing, China; 4 Singapore Eye Research Institute, Singapore National Eye Center, Singapore, Singapore; 5 Stanley Manne Children’s Research Institute, Ann & Robert H. Lurie Children’s Hospital of Chicago, Chicago, IL USA; 6 Netspective Foundation, Inc., Silver Spring, MD USA; 7 Division of Endocrinology, Department of Internal Medicine, College of Medicine, King Saud University, Riyadh, Saudi Arabia; 8 Division of Endocrinology, Diabetes & Metabolism, The Johns Hopkins University, Baltimore, MD USA; 9 Sutter Health, Emeryville, CA USA; 10 Mills-Peninsula Medical Center, Burlingame, CA USA; 11 Palo Alto Medical Foundation, Sutter Health, Palo Alto, CA USA; 12 Boston Children’s Hospital, Harvard Medical School, Boston, MA USA; 13 School of Medicine, Johns Hopkins University, Baltimore, MD USA; 14 Department of Computer Science and Engineering, Shanghai Jiao Tong University, Shanghai, China; 15 MOE Key Laboratory of AI, School of Electronic, Information, and Electrical Engineering, Shanghai Jiao Tong University, Shanghai, China; 16 Department of Genetics, Stanford University, Stanford, CA USA; 17 Department of Ophthalmology, Yong Loo Lin School of Medicine, National University of Singapore, Singapore, Singapore; 18 Diabetes Research Institute, Mills-Peninsula Medical Center (Sutter Health), San Mateo, CA USA

**Keywords:** Artificial intelligence, Human oversight, Machine learning, Transparency, Trust

## Abstract

Artificial Intelligence (AI) has the potential to impact healthcare across multiple domains. In diabetes, a complex chronic disease affecting 600 million people globally, AI is already being used from primary care to tertiary specialist care to reduce patient and clinician burden. However, for medical AI to be widely implemented and applied specifically to diabetes, such stakeholders as patients, clinicians, healthcare administrators, regulators, and AI developers will need to establish trust in this technology. Building trust is a balancing act depending on individual priorities of stakeholders which may not necessarily align. Both probabilistic outputs and “top-choice only” outputs are used in medical AI. To achieve trust in AI for diabetes care, it will be necessary to move beyond expecting only single, deterministic outputs and to establish clear standards for medical AI provenance and performance. This article presents priorities for each of the various stakeholders if they are to develop trust in medical AI and their responsibilities for contributing to the establishment of trust in medical AI. For a medical AI system to be trustworthy, six key attributes must be incorporated including accuracy, reproducibility, privacy/security, transparency, human oversight, and fairness. We present practical methods to achieve each of these six attributes of trustworthy medical AI prioritizing diabetes that are important for all stakeholders.

## Introduction

The integration of artificial intelligence (AI) into mainstream healthcare offers substantial opportunities. However, a fundamental principle for any new healthcare technology to be successful is the need for trust by the key stakeholders in healthcare, including patients, clinicians, and healthcare system stewards, such as administrators and regulators.

About 38 million people in the United States have diabetes, and 98 million have prediabetes [1]. Diabetes is the seventh leading cause of death in the United States [2], and the most recent comprehensive estimate puts the annual economic cost of diabetes at about $413 billion [3]. Diabetes is an especially suitable exemplary disease for AI in digital health because it is highly data-rich, with continuous information from glucose monitoring, insulin delivery systems, and connected apps, and it has a long history of adopting digital technologies in routine care. It is also common and clinically significant, and already a leading use-case for AI-enabled tools such as automated insulin dosing, risk prediction, and digital therapeutics, making it a practical domain in which to demonstrate real-world AI impact. In addition, diabetes management is anchored in clear, quantifiable outcomes, such as hemoglobin A1c (HbA1c), time-in-range, glycemia risk index, and hypoglycemia rates. These metrics enable rigorous measurement of how AI systems affect both day-to-day control and long-term complications. As an example, for adults and children with type 1 diabetes using automated insulin delivery (AID) systems (many of which will soon be managed by AI), trust is a necessary although not sufficient cornerstone of acceptance of this technology, given the immediacy and severity of risks if the system malfunctions. Recent increasing adoption of AID systems in clinical care can be attributed primarily to the glycemic benefits and accumulation of real-world evidence, rather than significantly greater trust as a specific driver of recent uptake [4, 5]. In this article, we present evidence about trust in AI applied to healthcare and AI applied to diabetes based on the four elements of the following analytic framework. First, we evaluate current evidence of trust in AI for health. Second, we identify the five key stakeholders who most need to trust AI for successful deployment. Third, we present six principles of Trust in AI that are necessary for all the stakeholders to have trust in the use of medical AI. Fourth, we present principles of Trust in AI that are necessary for AI applied to diabetes.

## Current evidence of trust in AI for health

Recent studies show that patient trust in AI is mixed. Surveys of patients about preferences have reported both greater [6] and lesser [7] trust in physicians who use AI to help make medical decisions. A recent study found that patients could not reliably distinguish AI from physician answers and low-accuracy AI advice was rated similarly or in some cases higher than doctors [8].

An AI model is likely to be more trusted if it has demonstrated generalizability to populations relevant to the patient being treated. Trust in medical AI can also be influenced by previous experiences that a particular patient or clinician has had with medical AI. Existing trust or distrust in the broader healthcare system and individual providers also influences how medical AI is perceived. Patients who have experienced discrimination or medical errors may be more hesitant to trust new technologies unless benefits are very clear. The opposite of AI distrust is a delusional or excessive trust in AI, which assumes the model is always correct or even capable of human judgement, and this belief has been named “AI psychosis.” Overall, patient trust in AI appears to be shaped more by individual experiences and the specific clinical context than by a pervasive skepticism.

In diabetes care, where digital health tools (e.g., continuous glucose monitors [CGMs]) are increasingly being used for personalized diabetes self-management, as well as supporting behaviors intended to improve the quality of glycemia, there are enormous opportunities for AI. In a survey of 418 people with diabetes, 82% of participants indicated a willingness to adopt AI-integrated wearable devices if recommended by healthcare providers [9]. Already, AID systems are being used by increasing numbers of people with type 1 diabetes and also adults with type 2 diabetes, but not the only—or always dominant—determinant of AID adoption and persistence, and thus rising population‑level use of pumps/AID cannot be directly interpreted as a linear signal of rising trust, indicating an increasing degree of trust in these systems. AI is likely to improve the efficiency of existing insulin delivery systems, especially if reinforcement learning is used, to provide a more personalized and fully automated closed-loop to glucose management.

## Key stakeholders for AI

**Fig. 1:**
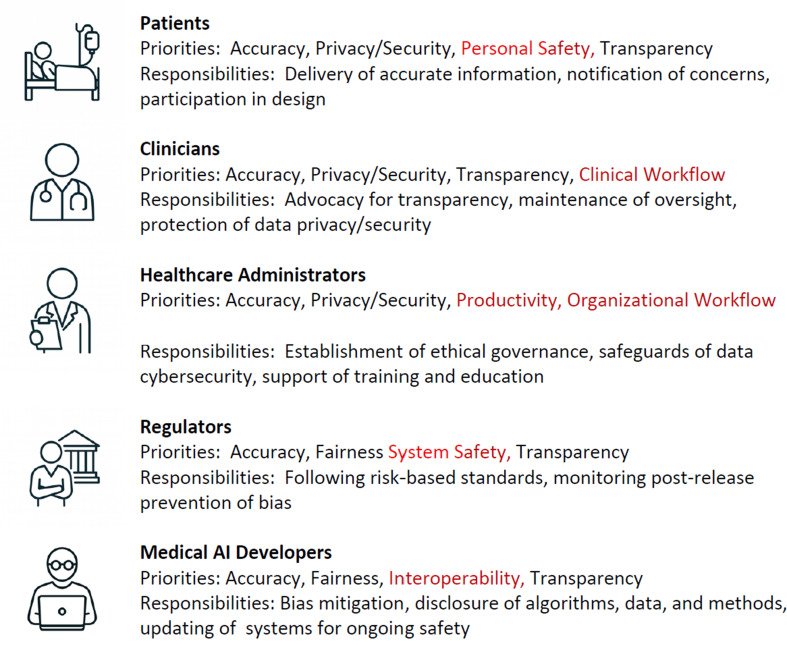
The priorities and responsibilities for contributing to the establishment of trust in medical AI. Legend: Top priorities and responsibilities are presented in alphabetical order for each stakeholder. Red font indicates a priority for that stakeholder. Abbreviations: AI, Artificial Intelligence

For a medical AI program to be successful, five key stakeholder groups—patients, clinicians, healthcare administrators, regulators, and AI developers—must place their trust in the program, guided by their own distinct priorities. These groups must also collaborate in the development, deployment, implementation, and monitoring of AI for this approach to healthcare to succeed. Priorities for trusting medical AI and three top responsibilities for contributing to the establishment of trust in medical AI for each of the five key stakeholders affected by medical AI are presented in Fig. 1. Whereas accuracy, transparency, privacy/security, and fairness are among the top four priorities on multiple lists, each stakeholder has at least one unique priority in its top four, including personal safety (patients), clinical workflow (clinicians), productivity (healthcare administrators), organizational workflow (healthcare administrators), system safety (regulators), and interoperability (AI developers).

Many patients appear to not fully trust medical AI, are skeptical of AI in terms of its accuracy and value, are fearful of its potential risks, and still prefer a human clinician over an AI-powered system, either acting autonomously or operating in assistance with the human clinician (“co-pilot”). In a recent survey, US adults were significantly less likely to trust, feel empathy toward or seek care from a physician who advertised using AI [6]. In a recent international survey, adults preferred AI implementation strategies that account for user demographics and health status, as well as explainable AI supported by physician oversight [6]. Explainable AI refers to users having access to processes and methods that enable them to comprehend and trust the results and outputs created by AI as opposed to the “black box” nature of many current AI models.

Clinicians have concerns about the accuracy, transparency, and accountability associated with using AI in their practices and their opinions about AI are also likely to influence patient acceptance of medical AI [7]. Clinicians also must have trust in the healthcare system to confidently deploy AI. They might worry that AI tools have been selected not for their clinical accuracy or to provide decision support, but instead to streamline workflows or improve profitability. A recent survey study found that clinicians using, compared to those not using, generative AI for verification or primary decision-making, had negative perceptions from their peers [10]. Furthermore, some clinicians whose day-to-day job involves providing pattern recognition (e.g., histopathologist and radiologists) are fearful of being replaced by AI. Healthcare administrators set policies and determine deployment of new technologies. When they provide oversight to the trustworthiness of AI’s capabilities, that accounts for all stakeholders’ interests, then they are likely to facilitate its adoption through supportive policies. Regulators establish and enforce standards that safeguard patient well-being and technology reliability through premarket validation and postmarket surveillance for accuracy, transparency, safety and ethical considerations. Finally, AI developers play an important role in building trust in medical AI, which requires not only technical expertise, but also attention to transparency, safety, ethics, stakeholder collaboration, and regulatory adherence.

## Barriers and solutions to stakeholder misalignment around trust in medical AI

Currently, the five stakeholder groups do not share the same degree of trust in medical AI, and their priorities and perspectives are often misaligned. Three cross-cutting types of barriers to alignment, related to engagement, communication, and workflow integration of AI-enabled systems, and corresponding solutions are summarized in Table 1.

**Table 1:** How alignment can be achieved or measured in real-world deployments to develop trustworthy AI: barriers and solutions

Barrier	Solutions
Potential discordance of priorities	• Patients may prioritize privacy/security • Healthcare decision makers may prioritize efficiency • Clinicians may prioritize diagnostic accuracy [11, 12]
Patients distrustful of Artificial Intelligence for healthcare	• Ongoing education to build trust • Plain-language summaries of AI decisions • Creation of visual dashboards showing trends rather than just numeric outputs • Analogies (e.g., “the AI works like an experienced assistant checking patterns in your glucose readings”) • Educational materials should be co-designed with patient advocacy groups to ensure cultural appropriateness and clinicians to ensure clinical relevance [13, 14]
Clinicians are concerned that AI could disrupt workflow, add to responsibilities, and increase total workload	• Consensus-building multi-disciplinary panels can allow clinicians to negotiate workflow adaptations and liability issues with other stakeholders • Education programs to improve clinician AI literacy and data interpretation skills • Focus on domains where clinical judgment and empathy, cannot be fully automated—such as patient counseling, nuanced diagnoses, and multidisciplinary team [15, 16]

## Principles of trust in AI

**Fig. 2:**
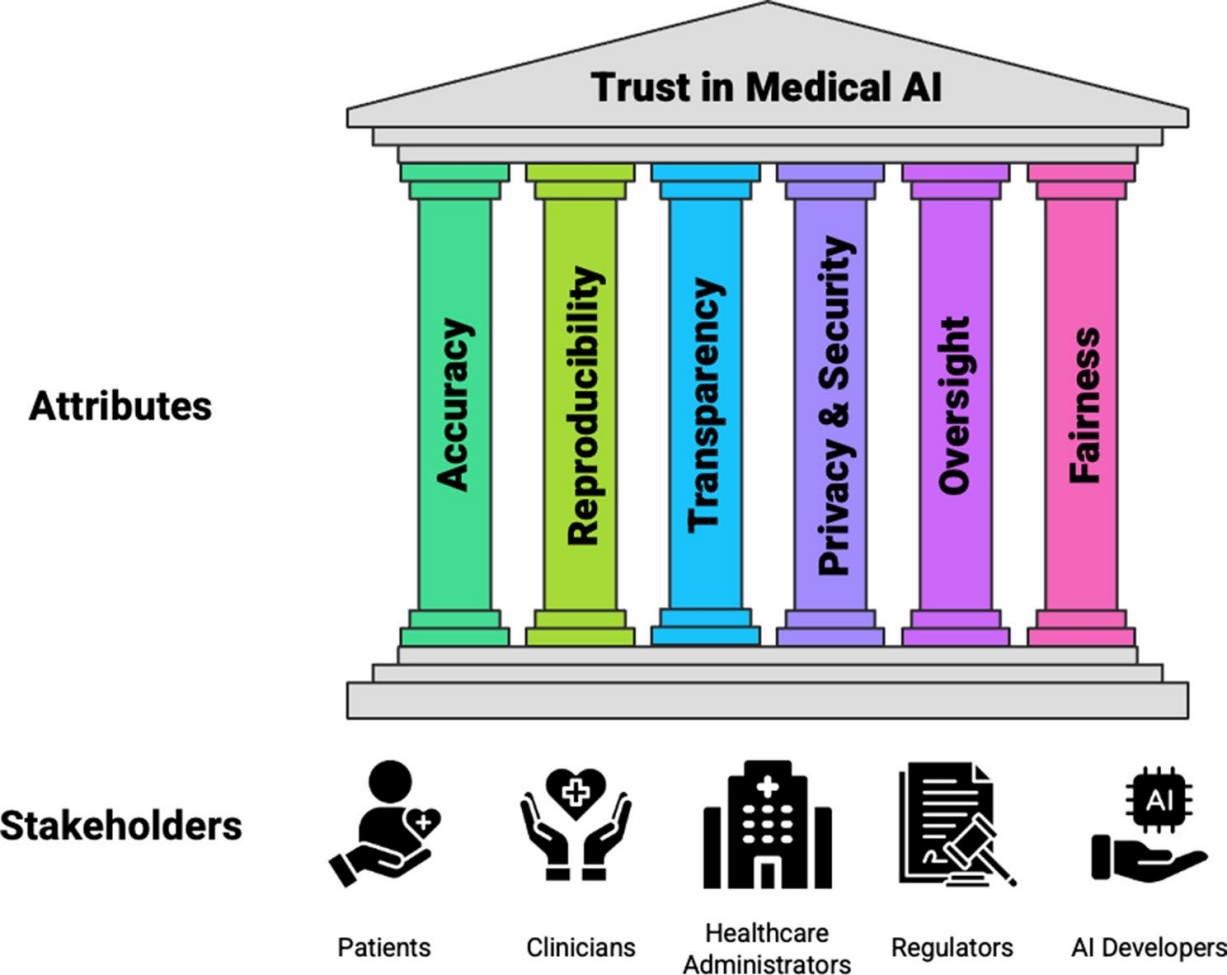
The six attributes and the five key stakeholders of trustworthy medical AI. This figure has been designed using resources from Flaticon.com. Abbreviations: AI, Artificial Intelligence

For all five key stakeholders involved with medical AI, creating trust in this paradigm requires creation of systems that are based on six attributes that are important to them. These attributes and stakeholders are presented in Fig. 2.

## Accuracy and AI

AI models can earn trust by transparently demonstrating robust, real-world accuracy—validated against clinician performance—while offering clear explanations. Demonstration of accuracy through randomized controlled trials and real-world evidence studies is therefore necessary for patients and clinicians to trust an AI model. If a tool cannot produce reliable and reproducible results, then neither patients nor clinicians will use it. A systematic review and meta-analysis of 83 studies validating AI models for diagnostic tasks published between 2018 and June 2024 revealed an overall diagnostic accuracy of 52% [17]. AI is particularly accurate in making diagnoses from radiology, pathology, and dermoscopic images, as well as from retinal photographs and smart stethoscope-generated heart sounds.

AI models have the ability to summarize and calculate based on their training data, but if the training data is flawed, non-representative, or outdated, then the model’s decisions will reflect those flaws. The selection of relative weightings and diagnostic cutpoints have a critical effect on outputs of medical AI models, and it is also important to account for a patient’s values and attitude about risk rather than accept a one-size-fits-all decision.

Using diabetes as the model disease, studies have shown that diabetic retinopathy screening algorithms trained on retinal fundus images from predominantly white patients underperform in more diverse populations. This data bias is an ongoing challenge in machine learning (ML). AI can also be used to classify metabolic subtypes of diabetes and prediabetes with data from CGMs so that prevention programs can be more targeted and personalized. ML models can diagnose various complications of diabetes including foot ulcers, chronic kidney disease, and neuropathy, in addition to retinal disease. Furthermore, AI-powered fully automated multihormone delivery systems not requiring meal announcements are now under development for diabetes.

Examination of performance of AI across diverse institutions, patient populations, and usage patterns based on evidence from randomized controlled trials will help uncover the generalizability of AI, and highlight potential biases. In a cross-sectional study of all 903 AI-enabled medical devices that had received FDA approval as of August 31, 2024, clinical performance studies were reported for only approximately half of them [18]. A reference population score can be used as an effective metric for assessing existing AI models. This metric aims to measure the diversity of the datasets used for training, validation, and testing during AI algorithm development. This includes evaluating the model against multiple populations and validation by expert consensus in real-world patient studies, to ensure adequate inclusion and explainability of the model performance. Addressing current gaps in documentation, evaluation, and post-market surveillance is essential to ensure stakeholders’ confidence in the accuracy of AI-enabled medical devices.

To gain trust, clinical AI tools must be tested rigorously on multiple populations and validated by expert consensus and results of real-world patient studies to maximize accuracy. However, technical success does not always translate across specialties or patient populations. Li et al. demonstrated that even state-of-the-art large language models (LLMs) struggle with specialized diabetes knowledge, which demonstrated limitations of AI when applied to domain-specific medical training. In a study testing (LLMs) on accuracy for the endocrinology board examination, ChatGPT and Bard only had 58% correct answers, with even lower scores in diabetes and diabetes technology.

## Reproducibility and AI

Reproducibility in AI means that independent users can replicate findings. This feature of AI is an essential condition for building and maintaining trust in these systems in healthcare and diabetes care. Reproducibility is necessary to verify claims about all the other features indicating the trustworthiness of an AI model. This feature of an AI model builds trust by the stakeholders, especially when the inner workings of a model are not fully transparent. AI systems that lack evidence of reproducibility potentially create risk for people with diabetes from misclassification or improper treatment, such as unsafe insulin dosing from an automated insulin delivery system.

To assess stability of classification of members within a dataset that could be referenced for an AI model, Ahlqvist et al. identified five diabetes clusters with different clinical phenotypes and outcomes in a Scandinavian population. A proportion of people with diabetes appeared to migrate between subtypes over time, which could limit the use of this subtyping approach for estimating long-term treatment response and prognosis. In contrast, Tanabe et al., developed an ML model to classify individuals with type 2 diabetes. After five years of follow up the subtype assignments persisted consistently [19]. This outcome demonstrated robust reproducibility over time of a subtyping model. Artificial Intelligence Ready and Equitable Atlas for Diabetes Insights (AI-READI) is a current project that is collecting and standardizing a large and diverse dataset for type 2 diabetes and is intended to facilitate research in AI reproducibility based on this dataset.

Providing model code and data in AI publications is fundamental to reproducibility, because this practice allows others to confirm results. In a recent review of 40 AI-based methods for diabetes risk prediction, only four articles made their code publicly available [20]. AI research will be more reproducible through more sharing.

## Privacy/security and AI

Trust in medical AI by both patients and clinicians depends on robust privacy/security protection. For a patient to trust the use of AI for their case, they must agree to provide medical AI systems access to large volumes of sensitive personal information. Clinicians must trust the privacy/security of AI systems to have the confidence to use these systems. Beyond confidentiality, security and resilience protect the integrity and availability of models and data. IEEE 2621 is the only consensus cybersecurity standard for diabetes devices with both performance and assurance requirements. It is recognized by the US FDA. Meeting this standard signals adherence to sound cybersecurity processes. The manufacturer of a connected insulin pen and companion diabetes management app, leveraging AI and analytics for real-time dosing support and personalized feedback, was once subject to a federal class action lawsuit after it was discovered that the app used tracking and analytics tools that collected and disclosed users’ health and personal information. On July 31, 2025, the Centers for Medicare & Medicaid Services announced a medical records interoperability initiative without a centralized government run database. This initiative will enable richer AI reference datasets, however, increased data liquidity also expands the attack surface and will require effective cybersecurity.

## Transparency and AI

Transparency is another prerequisite for building trust. Explainability is a powerful tool for building trust in AI, particularly in healthcare where AI output can have major consequences or require regulatory scrutiny. Users are more likely to trust AI systems when they understand how decisions are made. For example, a voice-based conversational AI application for explaining insulin dosing has helped patients with type 2 diabetes titrate basal insulin at home and achieve improved glycemic outcomes [21]. Transparent AI can mean both transparency in (1) how AI tools make decisions and (2) how AI tools are governed (i.e. data sources, algorithm choices, and auditing practices for performance and bias). Even the most accurate AI model can be met with skepticism if clinicians and patients do not understand how it works. Many ML models often operate as “black boxes,” meaning they generate outputs without clearly explaining the decisions they made to get there. This transparency issue is a serious barrier to adoption. Patients and clinicians both consistently report discomfort with non-transparent AI systems. However, there is often a trade-off between complexity and explainability.

Researchers and practitioners working with medical AI are increasingly focused on explaining the decisions made by AI applications. An emerging field called explainable AI (XAI) looks to promote trust in AI models by designing AI systems with explanations of specific factors that contribute to their AI-derived recommendation. By providing insight into the reasoning steps, XAI allows AI stakeholders to verify results, and provides insights into the accuracy, reproducibility, and fairness of AI-driven decisions. Current explainability techniques can produce descriptions of how the AI system works in a general sense but are often not fully informative regarding individual decisions. XAI, compared with standard AI, increases clinicians’ trust especially when explanations are clear, and relevant to clinical practice [21].

Transparency is a key issue not just with respect to how the models driving the AI works in the clinic, but in what the process was of building and validating the models. Concerns related to proprietary model architectures and training data add another layer of complexity toward making AI transparent. Industry-developed clinical decision support tools and large language models are often proprietary, and it is unclear what biases and values may be embedded in their decision-making processes.

## Human oversight and AI

The proper use of AI requires human oversight to identify errors and build trust. Patients often express a need for oversight from a physician to create trust. For clinicians, AI oversight means defining who holds liability for the outcomes of AI-generated recommendations. When an AI system relies on inaccurate data or flawed methods, its recommendations can mislead clinicians and ultimately compromise patient care. In such instances, if an AI system serves as a decision-support tool, then the physician might be held responsible as the final decision maker who is expected to verify the AI outputs. Healthcare organizations or decision makers may share liability if they deploy untested or unsafe AI tools, fail to adequately train staff, or do not put in place sufficient oversight of AI-driven decisions. Developers of AI models can also be liable if the system is flawed. Guidance is needed to determine the fair apportionment of responsibility for outcomes. Patients, who want greater clinical oversight of medical AI, and clinicians who want greater protection from liability from erroneous medical AI, will both have greater trust in medical AI if clinicians can be formally trained in how to use AI, when to trust AI, and when not to automatically trust AI. The reason that AI cannot currently replace clinicians is because clinicians have the ability to interpret data, maintain situational awareness, and draw from their unique clinical that comes with experience. These capabilities can surpass those of an AI algorithm developed using a dataset that may lack key historical or examination factors. Specialist clinicians often have deep knowledge of topics that will take a long time for AI to match.

Human oversight, also known as human-in-the-loop, is often (but not always) helpful in AI-assisted diabetes care because although AI models can analyze data, they may lack the clinical judgment necessary for individual patient needs. For adults and children living with diabetes and using insulin, they already have to make decisions related to the dose and timing of a medicine which, used inappropriately, can result in serious adverse outcomes. People with diabetes must understand the logic of AI-based support to fully trust this technology. When an AI-enabled prescription advisory tool for dosing insulin was introduced in Singapore, some clinicians expressed concerns about occasional contradictions between the AI-recommended dose and their own professional judgment. Human oversight is also important for recommending personalized settings in an AID system where this oversight is important for validating settings and addressing adverse events and edge cases.

To improve oversight of AI models, clear frameworks for liability, and standards for data quality are needed. Furthermore, post market surveillance is needed for performance because over time, performance drift (where the device’s predictive accuracy may decrease or shift because of new data patterns), exposure to new data types, or unanticipated clinical scenarios can occur. Thus, even when an AI model is initially well explained, real world data can diverge from training data, so two essential post-market processes to address these risks include ongoing monitoring for drift and adjustment of the AI model, i.e. recalibration, if needed.

## Fairness and AI

Medical AI models must be built on fairness to gain the trust of stakeholders. AI systems embodying fairness ensure that all patients receive the same quality of care and avoid bias. Fairness in AI builds trust to a similar extent for patients with diabetes as other diseases, but more dedicated AI-powered systems are now increasingly available to support people with diabetes and prediabetes though interpretation of CGM data, delivery of digital therapeutics, and recommendations of insulin dosing. Fairness for diabetes AI models can be assessed through the use of diverse diabetes datasets and independent validation with diabetes registries. For an AI system that creates patient-facing material, it will be important to ensure that the system takes into consideration challenges related to health and digital literacy as well as cultural congruence to ensure fairness.

In many communities, historical mistrust of medical institutions and perceived bias have weakened confidence in the healthcare system. In such cases, medical AI—when designed transparently and equitably—may improve relationships with the medical profession by offering consistent, impartial, and data-driven decision-making. Building on this potential involves ensuring outputs are delivered in culturally resonant formats, available in local languages, so that AI becomes a bridge rather than a barrier between patients and providers. A governance program promotes fairness and trust in medical AI by ensuring that AI systems serve all patients. According to a recent survey of health systems, 88% use AI, but only 18% have a mature governance structure [22]. Greater adoption of trustworthy AI governance by health systems is needed.

## Principles of trust applied to AI models for diabetes

Principles of achieving trust for AI used for diabetes are similar to principles for other medical purposes, but specific factors must be considered that are important for people with diabetes. In some instances, an AI model might have to be tuned to reflect a tradeoff between competing attributes, such as achieving greater accuracy at the expense of privacy/security, and vice versa. Table 2 presents 18 principles for promoting trust in AI systems for diabetes. Three practical methods to achieve each of the six attributes of trustworthy medical AI that are important for all stakeholders are listed in Table 3.

**Table 2:** Principles for achieving trust in AI systems using diabetes as a model disease

Principle	Description
Accuracy	• Data for an algorithm must derive from multiple patient groups to include populations that have been under-represented • Robust predictive accuracy close to ground truth must be demonstrated with data available in the public domain • The system must be validated for specific subgroups within a disease, e.g., type 1 vs. type 2 vs. gestational diabetes
Reproducibility	• Model code and validation datasets should be made available within AI publications to allow others to attempt to reproduce conclusions • Large diverse datasets should be shared (taking into consideration privacy/security concerns) to minimize bias wherever possible to facilitate fair head-to-head comparisons • Limited studies of AI used for predicting diabetes subtypes and future complications must continue to show reproducible outcomes over time
Privacy/Security	• Secure privacy/safeguards require accepted standards • Robust interoperability across devices is necessary • Engagement with digital health tools, such as CGMs, diet logs, and step counters, may decrease if there is fear of a data breach
Transparency	• Model output, method, assumptions, and data sources must be clearly explainable to stakeholders – patients, clinicians and systems • An algorithm can be validated against other datasets using accepted comparative methods • Characteristics of patient data used in a diabetes AI model are available, including age of onset of diabetes, BMI, laboratory data (e.g., HbA1c, antibody status), diabetes education, technology use, etc.
Human Oversight	• The AI System must be shown to integrate into the clinical workflow, including electronic health records • AI systems should support and enhance – not replace - human decision-making • An AI insulin dosing system can learn based on changes in insulin sensitivity, dietary/exercise preferences, and pharmacoadherence
Fairness	• Data for AI should be collected from all potentially affected groups and all groups should be accounted for in the AI algorithm to maximize trust • AI models must minimize biases and include an explanation of where data is missing from reference datasets • AI tools can analyze retinal images to detect early diabetic retinopathy, but retinal image features—and thus AI accuracy—can vary by ethnicity because of differences in fundus pigmentation and disease patterns

Legend: Abbreviations: AI, Artificial Intelligence; AID, Automated Insulin Delivery; BMI, Body Mass Index; CGM, Continuous Glucose Monitor; HbA1c, Hemoglobin A1c

**Table 3:** Practical methods to achieve six attributes of trustworthy medical AI that are important for all stakeholders

Attribute	Practical Methods
Accuracy	A public “trust scorecard” is a tool for communicating performance and trustworthiness. This simple, traffic-light style dashboard could show: • Accuracy compared to clinician benchmarks (e.g., green/yellow/red) • Diversity of training data (percentages by key demographic groups) • Date of last independent audit • Known limitations or contraindications • Scorecards can be displayed in patient portals, clinician dashboards, or regulatory filings to provide at-a-glance trust indicators
Reproducibility	A parallel multicenter testing program of the reproducibility of an AI tool • Testing of models will occur at independent clinics who will evaluate the same model on local patient datasets or shared synthetic datasets • Performance summaries can be published • This distributed validation approach builds confidence that performance is consistent across environments, not just in controlled trials
Privacy/Security	A discussion of privacy/security in plain language can explain issues beyond formal compliance with legal and engineering privacy/security standards. • A statement that the AI does not store identifiable information • An explanation of how anonymous pattern learning used in AI works, and how it protects a person’s medical story with recognition that it is important to monitor closely and continually improve safety rules to prevent anyone from figuring out a patient’s identity • An explanation that AI makes generalized predictions from population-level anonymized data and can apply these predictions to make individual outputs from patterns in an individual’s medical information
Transparency	An AI model card for the lay public, similar to a nutrition label on food, can be posted in patient portals or clinician tools to improve transparency without requiring technical literacy. Labels can list at-a-glance: • Purpose of the AI model • Data sources used • Known limitations and biases • Date of last performance review
Human Oversight	A two-tier review system to provide practical oversight to preserve efficiency while ensuring human judgment remains central where the AI is less certain: • High-confidence recommendations – approved automatically but logged for audit • Low-confidence or novel cases – flagged for manual review by a clinician
Fairness	An inclusive community program for all community stakeholders from the very beginning of developing an AI program • Focus groups with members from all communities • Collaboration with community-based organizations or leaders who already have the community’s trust • An ongoing feedback mechanism

Abbreviations: AI, Artificial Intelligence

## Conclusion

Trust is foundational to the successful implementation and continued use of AI in healthcare. We present the integration of trustworthy AI into diabetes care as an exemplar condition to illuminate principles applicable to a broad spectrum of diseases. Six core features of medical AI that are necessary for trustworthiness include accuracy, reproducibility, privacy/security, transparency, human oversight, and fairness. For medical AI models to become genuinely trustworthy, through development, deployment, and implementation, sustained attention to all six of these core features of AI is vital.

## Data Availability

Not applicable.

## Abbreviations

AI: Artificial intelligence
AID: Automated Insulin Delivery
BMI: Body Mass Index
CGM: Continuous Glucose Monitor
HbA1c: Hemoglobin A1c
LLM: large language model
ML: Machine learning
XAI: Explainable Artificial Intelligence
